# Comparison of flow velocity in ophthalmic artery between glaucomatous and normal subjects

**Published:** 2019

**Authors:** Uma Sharan Tiwari, Mohini Singh, Ankita Aishwarya, Akshara Gupta, Kirti Chhabra

**Affiliations:** * Department of Ophthalmology, Gajra Raja Medical College, Gwalior, India; ** Comprehensive Ophthalmology, Mahatma Eye Institute, Nagpur; *** Department of Radiology, Gajra Raja Medical College (G.R.M.C), Gwalior, India

**Keywords:** peak systolic velocity, end diastolic velocity, resistivity index, pulsatility index, flow velocity, ophthalmic artery, POAG, NTG

## Abstract

**Purpose:** To study the hemodynamic parameters in ophthalmic artery (OA) using color Doppler imaging in subjects with primary open-angle glaucoma (POAG), normal-tension glaucoma (NTG) and age matched normals.

**Methods:** Sixty-eight eyes of 68 subjects (41 males and 27 females) constituted material for this prospective observational study. They were divided into three groups; Group A had 24 patients with POAG, Group B had 18 patients with NTG and Group C had 26 normal subjects. They underwent CDI of OA. The outcome variables were peak systolic velocity (PSV), end diastolic velocity (EDV), resistivity index (RI) and pulsatility index (PI). Data were compiled and analyzed using one-way ANOVA analysis.

**Results:** The mean ± SD age of POAG patients, NTG patient and normal subjects was 59.95 ± 7.35, 58.11 ± 9.97 and 57.73 ± 5.39 years, respectively. The mean intra ocular pressure (IOP) was 29.8 ± 5.0, 15.9 ± 2.4 and 16.6 ± 1.7mm Hg in Group A, B and C, respectively. In group A, the mean PSV, EDV, RI and PI were 18.2 ± 3.80, 3.71 ± 1.40, 0.93 ± 0.12 and 2.8 ± 0.42. In group B, 26.6 ± 1.72, 4.93 ± 1.32, 0.84 ± 0.02 and 1.32 ± 0.20 and in group C, 35.4 ± 3.04, 8.08 ± 0.69, 0.77 ± 0.03 and 1.80 ± 0.17, respectively. All the values were found to be statistically significant (p < 0.05). Lower PSV and EDV were found in POAG and NTG patients, while RI was higher than in normal subjects.

**Conclusion:** The hemodynamic parameters are significantly affected in POAG and NTG patients. The PSV and EDV are decreased and RI is increased. EDV is more sensitive for the assessment of hemodynamic changes.

## Introduction

Glaucoma, a chronic progressive optic neuropathy, is the second leading cause of blindness worldwide [**1**]. Intraocular pressure (IOP) is known to be a major causative risk factor. Vascular factors have also been implicated in the pathophysiology of glaucoma [**2**]. The association of glaucoma with vascular diseases like diabetes, hypertension, and migraine, has been reported [**3**-**5**]. The association of glaucoma with specific circulatory events such as systemic hypotension and peripheral vasospasm indicates that vascular factors play an important role in the pathogenesis of glaucoma [**6**,**7**]. The recent application of Color Doppler Imaging (CDI) to the orbit potentially allows us to measure the velocity in the central retinal artery (CRA) and in the short posterior ciliary arteries (SPCAs) [**8**]. 

CDI allows information about the flow of blood to be superimposed in color on a B-mode gray-scale ultrasound image, which enables the direct visualization of specific vessels that can be interrogated to produce a spectral waveform. Flow velocity, known as Doppler shift frequency or simply, true Doppler shift, is strongly dependent on the insonation angle i.e. the angle between the emission sound beam and flow direction at sampled artery [**9**]. Maximum velocity is produced when Doppler angle is 0° and the sound beam is parallel to the direction of blood flow [**10**]. Peak systolic velocity (PSV) and end diastolic velocity (EDV) can be measured from the spectral display. The resistive index (RI) can be calculated according to the method of Pourcelot as RI = PSV – EDV divided by PSV [**11**].

In this prospective observational study, we used CDI to assess the hemodynamic parameters in Ophthalmic Artery (OA) in patients with Primary open-angle glaucoma (POAG), normal-tension glaucoma (NTG) and age matched normal subjects.

## Subjects and Methods

This prospective observational study was conducted in our center with collaboration of the Department of Radiology between April 2017 and May 2018. Sixty-eight subjects (41 males and 27 females) were included in the study. They were divided into three groups. Group A had 24 patients with bilateral POAG, Group B had 18 patients with bilateral NTG, and Group C had 26 normal subjects. 

Patients included in group A or B were over 18 years old, already diagnosed with POAG (group A) or NTG (group B), having no other ocular disease except for glaucoma. All patients with a history of systemic diseases with ocular involvement like diabetes and hypertension, ocular trauma, history of intraocular surgery (except for cataract surgery) and history of eye disease other than mild refractive error (<6/ 12) were excluded from the study. For group C patients with cardiovascular disease, diabetes mellitus, systemic hypertension, or hypotension, migraine, or vascular disease were not included in this study. Smokers, patients on systemic medication affecting blood flow, high myopia, and patients with media opacities were also excluded from the study.

POAG was diagnosed in patients who had reproducible localized visual field defects (VFD) and glaucomatous optic nerve damage with multiple intraocular pressure (IOP) readings over 21mm Hg [**1**]. NTG was diagnosed in patients who had reproducible localized VFD and glaucomatous optic nerve damage with multiple IOP readings lower than 21mm Hg [**1**]. The eye with greater glaucomatous damage was chosen in glaucoma patients, being randomly selected in the healthy individuals.

The procedures were in accordance to the Declaration of Helsinki and the International Code of Medical Ethics and were approved by the institutional ethical review committee. Each subject was required to sign an informed consent statement before enrolling in the study and prior to any study measurements were taken.

**Fig. 1:**
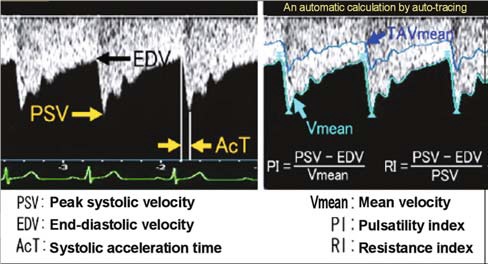
Parameters for Doppler evaluation of blood flow patterns

All CDI studies were performed on a Prosound Alpha 6 (Hitachi Aloka Products) ultrasound machine by a single experienced sonographer who was unaware of the subject’s clinical status. High frequency linear probe of 1-15 MHz was used. All examinations were carried out in the supine position with eyes closed. Blood flow in the retro bulbar orbit was detected by production of color pixels on visual display unit. In general, optic nerve was identified using a B scan, which is an important landmark for the detection of OA. The OA is situated either above or below the optic nerve into the posterior orbit before passing forward in the nasal orbit in a horizontal plane slightly superior to that of the optic nerve [**12**]. The outcome variables of this study were the following basic hemodynamic parameters: peak systolic velocity (PSV), which is the highest velocity of blood flow during the systolic phase of cardiac cycle, end diastolic velocity (EDV), which is the velocity of blood flow at the end of diastolic phase of cardiac cycle, resistivity index (RI), which is derived as RI = PSV-EDV/ PSV, pulsatility index (PI), which is derived as PI = PSV-EDV/ MFV, where MFV is the mean blood flow velocity (**Fig. 1**). 

The parameters of RI and PI represent the degree of distal resistance of vessels. RI focuses mainly on peak systolic resistance, but PI represents the resistance of the whole cardiac cycle. 

**Fig. 2:**
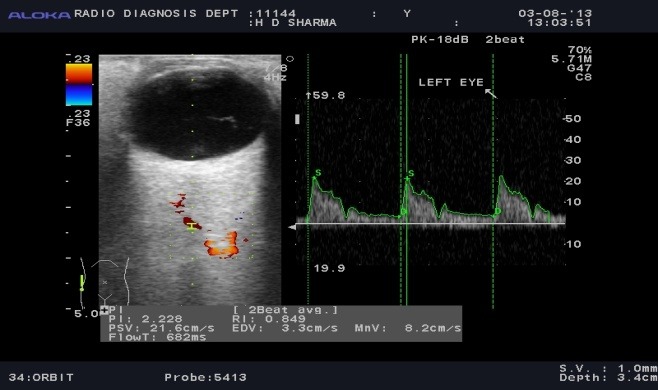
Colour Doppler photograph of an age matched healthy volunteer

**Fig. 3:**
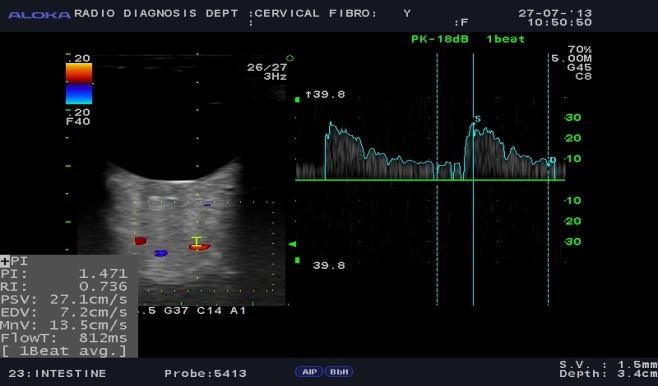
Colour Doppler photograph of a case of primary open angle glaucoma

**Fig. 4:**
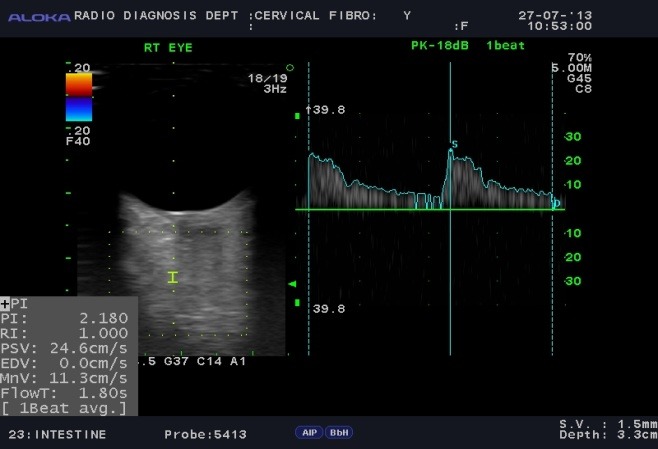
Colour Doppler photograph of a case of normal tension glaucoma

Color Doppler showing spectral waveform pattern in ophthalmic artery in different conditions was observed in normal subjects (**Fig. 2**), POAG (**Fig. 3**), NTG (**Fig. 4**). 

Descriptive statistics presented as mean ± SD. Baseline differences between groups were assessed by Student’s t-test. Differences between groups (pre and post treatment) were determined using one-way ANOVA analysis. P ≤ 0.05 was considered the level of significance.

## Results

The mean age of POAG patients, NTG patient and normal subjects was 59.95 ± 7.35, 58.11 ± 9.97 and 57.73 ± 5.39 years, respectively. There was no statistically significant difference between these age distributions groups. The mean intra ocular pressure (IOP) was 29.8 ± 5.0, 15.9 ± 2.4 and 16.6 ± 1.7 mm Hg in Group A, B, and C, respectively. The hemodynamic parameters used for study were quantitative PSV, EDV, PI, and the RI. In group A, the mean PSV, EDV, RI and PI were 18.2 ± 3.80, 3.71 ± 1.40, 0.93 ± 0.12 and 2.8 ± 0.42, respectively. In group B, the mean PSV, EDV, RI and PI were 26.6 ± 1.72, 4.93 ± 1.32, 0.84 ± 0.02 and 1.32 ± 0.20, respectively. In group C, the mean PSV, EDV, RI and PI were 35.4 ± 3.04, 8.08 ± 0.69, 0.77 ± 0.03 and 1.80 ± 0.17, respectively. All the values were found to be statistically significant. p value of PSV was < 0.0001, EDV p < 0.007 and in RI it was < 0.0001 (**Table 1**).

**Table 1:** The hemodynamic parameters among three groups (Values are expressed in terms of Mean ± SD)

Group	IOP (mmHg)	PSV (cm/ sec)	EDV (cm/ sec)	RI	PI
Group A (POAG)	29.8 ± 5.0	18.2 ± 3.80	3.71 ± 1.40	0.93 ± 0.12	2.8 ± 0.42
Group B (NTG)	15.9 ± 2.4	26.6 ± 1.72	4.93 ± 1.32	0.84 ± 0.02	1.32 ± 0.20
Group C (Control)	16.6 ± 1.7	35.4 ± 3.04	8.08 ± 0.69	0.77 ± 0.03	1.80 ± 0.17
*Peak systolic velocity = PSV, End diastolic velocity = EDV, Resistive index = RI, Pulsatility index = PI*					

The blood flow velocities were found to be reduced in glaucomatous subjects (both POAG & NTG) and resistivity indices were increased as compared to the control group. All the values were statistically significant (p<0.005).

## Discussion

The hemodynamic parameters are significantly affected in POAG and NTG patients. The PSV and EDV are decreased and RI is increased. EDV is more sensitive for the assessment of hemodynamic changes. 

With the mechanisms of glaucoma still so poorly understood, CDI may play a role in the hemodynamic pathophysiology of the disease, as well as in evaluating the effects of various medical and surgical therapies [**13**-**17**]. OA seems to be the most suitable vessel to investigate vascular aspects of Glaucomatous Optic Neuropathy (GON) [**18**]. Studies have confirmed that measurements of the hemodynamic variables of this vessel are reproducible and more reliable than the SPCAs and other vessels [**19**,**20**]. Although an indirect measure of blood circulation, the CDI can be used as a device to measure hemodynamic parameters of retro bulbar vessels. Hence, we studied the hemodynamic parameters of healthy and glaucomatous eyes in OA using CDI. We compared the findings of our study with the ones in related articles to draw conclusions (**Table 2**,**3**). 

**Table 2:** Hemodynamic parameters in POAG patients compared to Normal subjects (Mean value in Normal: mean value in POAG)

STUDIES	PSV	EDV	RI	PI
Akarsu C et al. (2004) [**21**]	Decreased (36.7):(34.7)	Decreased (12.57):(10.17)	Increased (0.65):(0.70)	-
Galassi et al. (2008) [**22**]	Decreased (28.01):(27.4)	Decreased (7.7):(6.8)	Increased (0.69):(0.74)	-
NC Sharma et al. (2006) [**23**]	Decreased (51):(25.85)	Decreased (23.85):(5.09)	Increased (0.55):(0.81)	-
Butt Z et al. (1997) [**24**]	Increased (30.8):(40.4)	Decreased (8.3):(7.8)	Increased (0.73):(0.81)	-
Pinto et al. (2012) [**25**]	Decreased (40.1):(33.6)	Decreased (7.35):(6.79)	No change (0.82):(0.80)	-
Martinez et al. (1999) [**26**]	Decreased (34.1):(31.2)	Decreased (9.2):(6.5)	Increased (0.73):(0.79)	-
Gherghel et al. (2000) [**27**]	Decreased (37):(35.25)	Decreased (7.9):(7.2)	Increased (0.78):(0.79)	-
Liu et al. (1998) [**28**]	Decreased (39.5):(34.3)	Decreased (11.15):(8.31)	Increased (0.72):(0.76)	-
Stalmans et al. (2009) [**29**]	Decreased (45.8):(33.3)	Decreased (10.4):(6.2)	Increased (0.78):(0.82)	-
Zeitz O, et al. (2005) [**30**]	Decreased (32.4):(27.5)	Decreased (7.31):(4.36)	Increased (0.77):(0.95)	Increased (2.01):(2.46)
Odunlami Olufemi Adeyinka et al. (2013) [**31**]	Decreased (37.6):(31.3)	Decreased (13.8):(9.1)	Increased (0.61):(0.70)	-
Present study	Decreased (35.4):(18.2)	Decreased (8.08):(3.71)	Increased (0.77):(0.93)	Increased (1.8):(2.8)
*Peak systolic velocity = PSV, End diastolic velocity = EDV, Resistive index = RI, Pulsatility index = PI*				

**Table 3:** Hemodynamic parameters in NTG patients compared to Normal subjects (Mean value in Normal: Mean value in NTG)

STUDIES	PSV	EDV	RI	PI
Butt Z et al. (1995) [32]	Increased (30.8):(31.5)	Decreased (8.3):(7.1)	Increased (0.73):(0.77)	-
Pinto et al. (2012) [25]	Decreased (40.1):(35.9)	No change	No change	-
Plange et al. (2006) [33]	Decreased (32.8):(30.8)	No change	No change	-
Present study	Decreased (35.4):(26.6)	Decreased (8.08):(4.93)	Increased (0.77):(0.84)	Increased (1.8):(1.32)
*Peak systolic velocity = PSV, End diastolic velocity = EDV, Resistive index = RI, Pulsatility index = PI*				

In all the above-mentioned studies, including ours, it has been observed that the hemodynamic parameters are significantly affected in glaucoma patients as compared to normal subjects, particularly the EDV and RI. Blood flow velocities were found to be significantly decreased whereas the resistivity index was found to be increased in all the glaucoma subjects including the NTG. 

PSV reflects the strength of vessel perfusion, whereas EDV reflects the blood perfusion of distal organs and is a sensitive indicator of increased downstream impedance [**34**]. RI is considered to reflect vascular resistance peripheral to the location where the measurement is made, but is not equivalent to vascular resistance because it depends on both vascular resistance and vascular compliance; only in high vascular compliance is RI an adequate measure of vascular resistance [**35**].

The possible mechanism of changed hemodynamic parameters in Glaucoma can be observed below.

In support of the relationship between these vascular changes and glaucoma, an increased correlation between ocular perfusion pressure (OPP) and retro bulbar blood flow velocities have been demonstrated [**36**]. Reduced blood flow velocities could be a sign of inadequate mechanisms of auto regulation in glaucomatous patients. This might also be due to increasing distal end resistance of the optic disc as a result of increased IOP. The POAG eyes with high IOP have significantly reduced ocular blood flow [**37**]. As reported, the increased RI may perhaps be caused by long standing vascular compromise in NTG and raised IOP in POAG [**38**]. 

The association of lower EDV with higher RI can be attributed to the increase in vascular resistance that affects diastolic blood flow velocity more than systolic velocity, which further aggravates the ischemia of organs [**39**-**40**]. Thus, EDVs are thought to be more sensitive for hemodynamic changes than the PSVs [**40**].

Due to short duration of follow up, glaucoma progression analysis could not be performed in our study. Thus, a correlation between hemodynamic parameters and progression of visual fields could not be established. 

**Financial support and disclosure**

None.
